# Numerical Models of the Connection of Thin-Walled Z-Profile Roof Purlins

**DOI:** 10.3390/ma14216573

**Published:** 2021-11-01

**Authors:** Přemysl Pařenica, Petr Lehner, Jiří Brožovský, Martin Krejsa

**Affiliations:** Department of Structural Mechanics, Faculty of Civil Engineering, VŠB—Technical University of Ostrava, L. Podéště 1875, 70800 Ostrava, Czech Republic; premysl.parenica@vsb.cz (P.P.); jiri.brozovsky@vsb.cz (J.B.); martin.krejsa@vsb.cz (M.K.)

**Keywords:** numerical analysis, Ansys, parametric study, roof purlins, thin-walled cold-rolled sections, Z-profiles

## Abstract

High thin-walled purlins of Z cross-section are important elements in steel wide-span structures. Their behaviour is influenced by many variables that need to be examined for every specific case. Their practical design thus requires extended knowledge of their behaviour for the possible configurations and dimensions. Numerical analysis verified by experimental investigation can thus enrich such knowledge. Numerical models have the advantage of repeatability and the ability to offer parametric changes. The parametric study presented shows a detailed description of a finite element model of thin-walled cross-sectional roof purlins connected to other roof elements. Models include various approaches to modelling bolt connection. Two schemes of purlins, with and without cleats, are presented. The results of different approaches in numerical modelling are compared with the results of a physical test on a real structure. The article shows a significant agreement in the case of specific approaches and points out the differences with others. The results can be helpful in terms of how to approach the modelling of thin-walled structures and the effective approach to experimental preparation.

## 1. Introduction

Thin-walled cold-rolled cross-sections (TWCC) are typically used in large-span halls. The use of TWCC has the main advantage of saving material, reducing construction costs and easier handling of individual structural elements [1,2,3,4]. On the contrary, the methodology used for design and assessment is more complicated. One of the few disadvantages of these elements may be the complicated design and assessment methodology, but also the lower resistance of the cross-section to off-axis loading and supports [5,6,7].

The analytical methods for solving the load capacity of thin-walled elements are the traditional methods of their assessment and are implemented in European standards and also in [8]. In EN 1993-1-3 [9], these methods are developed for the assessment of thin-walled sections by the effective width method. The essence of these standard assessments is summarized and described in detail, for example [10], where procedures for the solution of various thin-walled structures are given, including computational examples. The reduced area method consists in designing elements from thin-walled sections for which stability effects are taken into account through reduced effective cross-sections, most often by the iterative procedure presented in [9].

The solution of various problems of thin-walled structures can be inspired by the design for steel storage, where thin-walled profiles are very often used [5]. Standards and articles related specifically to steel storage show that there is a need to address section and load asymmetry [11], which was taken into account in the present research by bending the stiffeners. Another aspect is then the stability of the cross-section in the erection condition [12], although that is not the domain of this study.

Due to the sensitivity of the connection, thin-walled structures are an important type of task for physical testing and numerical modelling, because only in this way can the actual behaviour of new types and combinations be perfectly captured [2,13,14]. An important area is the evaluation of joints reinforced with local reinforcements, stability elements, double profiles or variable cross-section sizes.

The presented work is focused on numerical modelling of the behaviour of high thin-walled purlins with a height of 300 mm at the point of connection to the supporting structure in two variants, without a cleat and with a cleat. The details of the placement of thin-walled purlins was first experimentally investigated [15] and, subsequently, various types of numerical models were prepared. The variants included different approaches to modelling the purlins themselves, but also different approaches to modelling the joints, and, finally, different material models were included. The results of numerical models are compared with the results of a physical test in the form of force-displacement diagrams.

### 1.1. Available Numerical Approaches

There are several approaches to solving thin-walled cross-sections using numerical methods. In general, these are the finite element method (FEM) [16] and the finite strip method (FSM) [17]. The difference between these methods lies in the definition of finite elements. In the FEM, the entire structure is divided into discrete elements. On the contrary, the FSM is a simplified version in which the structure is divided into strips along the length of the element, which results in simplification and acceleration of the calculation. As an alternative to the FSM method, Schafer and Peköz developed the Direct Strength Method (DSM), which is presented in [18]. The DSM method considers the effective stress derived from the critical yield strength, compared to the original FSM, where the effective width of the elements is determined by an iterative calculation. The DSM method has been validated by an extensive research [19,20]. The FEM is, however, more universal in terms of geometry and material properties’ distribution of studied problems. Thus, it was selected for the presented works.

### 1.2. Overview of TWCC Analyses Worldwide

There are several procedures in which designs and assessments are made based on physical experiments and numerical modelling. These surveys most often refer to selected design systems of a specific manufacturer or at least design solutions, such as overlap [4,21], for which detailed methodology is not given in the Eurocodes. The issue of thin-walled profiles was investigated, for example, by a scientific team from the University of Corua, Spain. Two basic types of design for the supportive area of Z-profile roof purlins were experimentally and numerically compared [22].

The above research shows that the alternative with the overlap is more durable and achieves higher rigidity. Another conclusion of the research resulting from the experimentally verified data presented is the finding that the distribution of bending moments when using a bending moments envelope does not correspond to the behaviour of a continuous beam, and therefore such a design would be dangerous. In their further research [23], the authors numerically analysed in detail the connection of purlins with a focus on the stiffness of the connection of Z purlins with overlap. Numerical models have demonstrated sufficient stiffness of the overlap of Z-profile pairs that are commonly used in Europe. These are semi-rigid joints that have sufficient rotational capacity and can be considered in the global numerical model as continuous beams.

In [22] can be found research in which the authors analysed the influence of longitudinal reinforcements in Z-rofiles and verified the behaviour of the profiles with a cleat against net bending stresses, pure shear, and then also a combination of these two stresses. Here the authors prepared physical experiments and detailed numerical models in software ANSYS. The result is an evaluation of the influence of longitudinal reinforcements on the load-bearing capacity and a statement that after the confrontation with the DSM method, it can be said that it is sufficiently accurate for estimates of shear and bending load capacity of purlins in a specifically selected configuration. Finally, the authors evaluated the interaction diagrams with the conclusion that, for Z purlin, it is appropriate to replace the circular interaction diagram with a trilinear one, which provides less conservative results and is, therefore, more effective from the point of view of optimization.

The analysis of the connection of purlins with overlap was also conducted by a team of authors from the University of Sydney in their work [24], where Z-profile joints were faced with procedures and designs according to Australian, New Zealand and North American standards. The authors analyse configurations where there is a combination of bending and shear, evaluate the behaviour for different lengths of Z-profiles with overlaps, and vary the states when the flanges of Z-profiles were interconnected using bolts. ABAQUS SW was used for numerical models. The numerical models were validated with the results of physical experiments. The output is an evaluation that the DSM is in some cases nonconservative for combined bending with shear and can lead to a poor estimation of the behaviour of the Z-profile with overlaps. In general, numerical modelling of steel structures increases knowledge of the problem [25,26], so there is no doubt about the reasons for large-scale parametric studies.

## 2. Experimental Program

**Figure 1:**
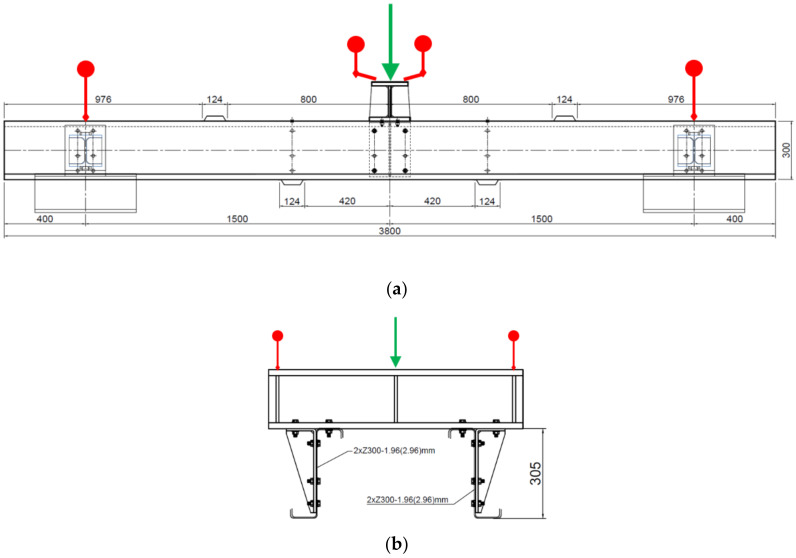
Scheme of the load test (**a**) a side view and (**b**) a cross-section of the location of the distribution segment.

The subject of the numerical study is the experimental program detailed in an earlier paper [15]. There was a special test set that imitated the gravitational load of other parts of the roof. Adverse effects that were not needed for the analysis were eliminated by experiment configuration. The test setup is schematically shown in Figure 1, which also shows the location of the displacement sensors and the location of the load loaded by the press.

**Figure 2:**
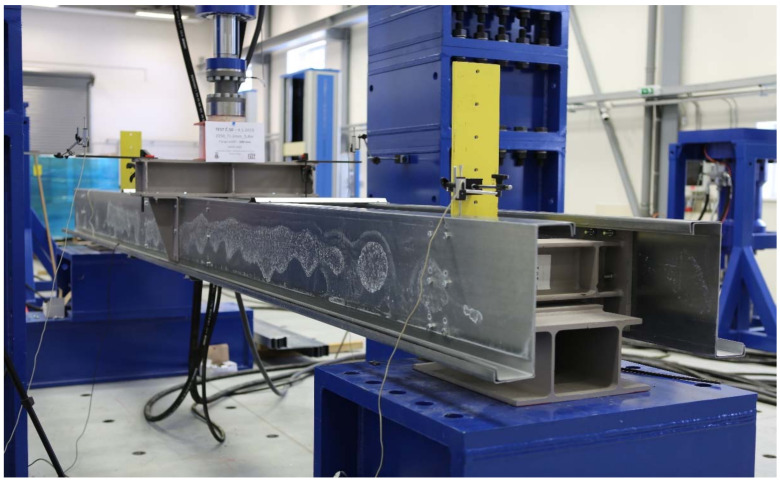
Experimental setup of TWCC in a hydraulic press machine.

The purlins were thus loaded in a way which is very close to the most common type of a load of every roof structure. Under load, combined stress occurs at the bearing point, both from the bend and from the pressure at the contact point of the thin-walled purlin flange and the main supporting structure. The test setup is schematically shown in Figure 1, which also shows the location of the displacement sensors and the location of the load loaded by the press. M12-4.6 bolts were used. The hole diameter was 13 mm. According to EN 1993-1-8 [27], the connections were rated as category A, which does not require prestressing, and therefore the bolts were hand-tightened. Figure 1. Scheme of the load test (a) a side view and (b) a cross-section of the location of the distribution segment. This figure also shows the arrangement of the stabilising partitions. The red markers show the position of the extensometers, and the green arrow shows the load point. The span between of the supports is 3000 mm. Above the supports, below the load and about 500 mm from the centre, there are reinforcing elements to prevent buckling. The reason for this is to try to evaluate purely symmetrical loading and the behaviour of the structure. The actual laboratory test setup is shown in Figure 2, where the arrangement of the individual parts of the experiment can be seen.

## 3. Numerical Modelling

**Figure 3:**
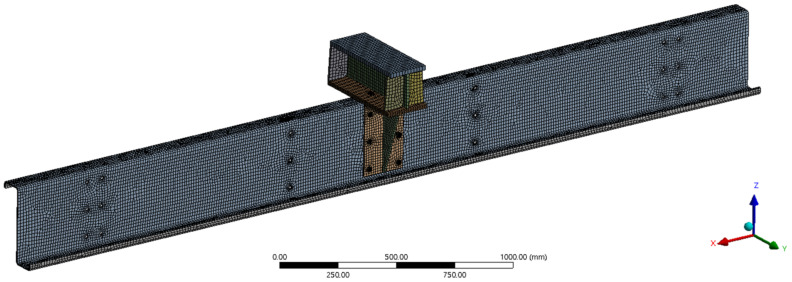
Experimental setup of TWCC in a hydraulic press machine.

The experiment was numerically re-modelled in the ANSYS 2020 R1 software [28] which is based on the FEM. Two sets were prepared, with and without the cleat. Due to the symmetry, only half of the load assembly was modelled, which was divided in the longitudinal axis of symmetry in the middle of the crossbar (see Figure 3). Some simplifications have been made in the numerical model as it is described below.

### 3.1. General Assumptions

All numerical models were prepared as complex spatial models and included non-linear geometric, structural, and material properties. The global model was created by connecting the individual components using an assembly approach implemented in the ANSYS Workbench project interface. For each component, its geometry was created in AutoCAD software and imported into the ANSYS. Subassemblies of finite elements of the model were created from such geometry and then were connected by contact elements. These subassemblies were merged into the final assembly, forming the main computational model. In it, the contact relations between all subassemblies were solved mostly through frictional contacts. Then the boundary conditions were applied.

There were two alternatives of models for purlins. The first variant was the model of purlins as solids using a solid-shell volume element. The second variant was the model of thin-walled purlins as shells using a shell-type element. Three types of bolt connections were also studied. The third variable parameter is the material model. Results including linear, bilinear, and multilinear stress-strain diagrams are presented. The system of nonlinear equations was solved by the quasi-Newton method.

### 3.2. Finite Element Types

**Figure 4:**
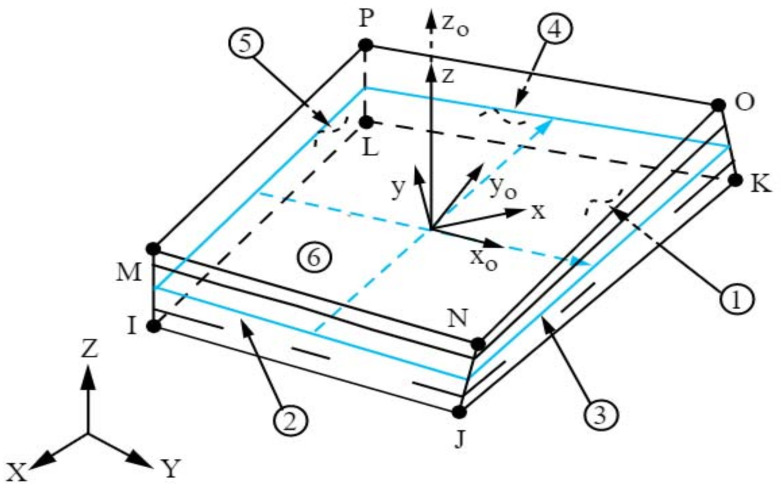
Schematic diagram of element SOLSH190 [28].

**Figure 5:**
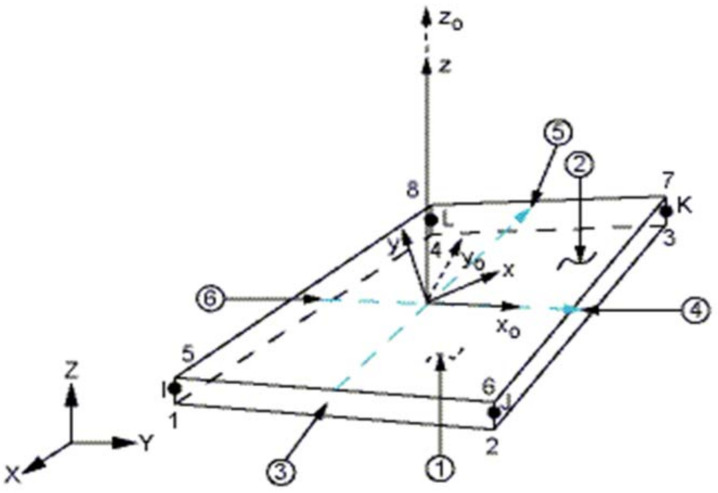
Schematic diagram of element SHELL181 [28].

The following two types of finite elements were chosen for the creation of finite element meshes. Solid-shell SOLSH190 (see Figure 4) and shell SHELL181 (see Figure 5) [28]. SOLSH190 is a 3-D volumetric 8-node element with three degrees of freedom at each node (UX, UY, UZ). The advantage of these types of finite-element elements is the possibility of efficient meshing on planar elements that are geometrically modelled as solids. However, the disadvantage is that its use requires the necessary simple topology of the element.

SHELL181 is a classic shell 4-node element with six degrees of freedom at each node. The advantage of these elements is that it is relatively easy to create a high-quality network even on topologically more complex surfaces. It is also easier to set the meshing parameters. The disadvantage may be the necessity to create an idealized geometry with a surface parallel to the centreline of the modelled structure, which is especially problematic when converting the geometry from a CAD model to a computer model.

**Figure 6:**
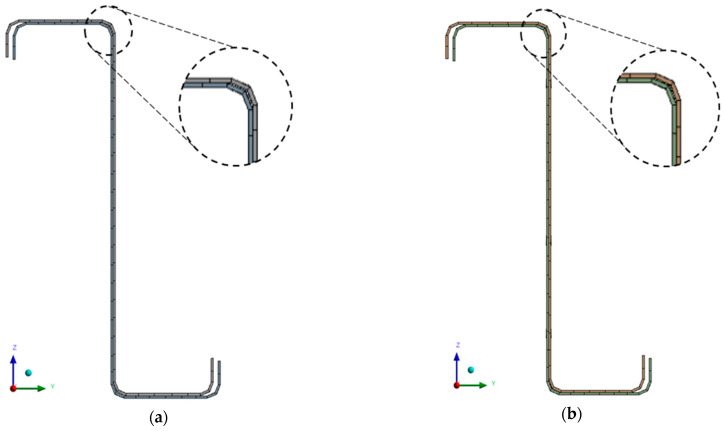
Comparison of finite element mesh of a pair of thin-walled purlins: (**a**) SOLSH190, (**b**) SOLID181.

The size of the finite elements was chosen to be the same for both types. The basic size of the elements was set to 15 mm. Furthermore, the division of the edges of the holes into 12 pieces around the perimeter was modified, which, among other things, led to a certain density of the mesh around the holes. A comparison of meshes consisting of solid-shell elements and shell elements is shown in Figure 6, from which there is virtually no significant difference in quality between them in terms of their described geometry.

### 3.3. Contacts Modelling

Non-linear contacts were set at the points of contact between the model parts to account for friction between the surfaces. The contacts are implemented using the TARGET170 and CONTA175 contact elements, which allow defining mutual contact pairs between two surfaces (face to face) and between a surface and an edge (edge to face). All contacts were set as frictional with a coefficient of friction value of 0.11 [29]. This value corresponds to the average value of the coefficient of friction between the steel surfaces.

**Figure 7:**
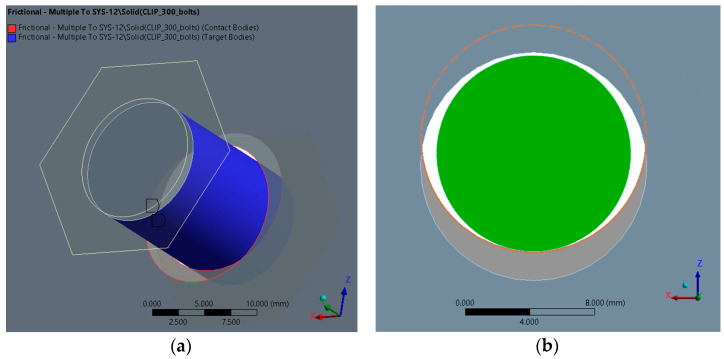
Contact pair between the holes of (**a**) the pair of Z profiles and (**b**) the bolt shank.

In the model, there are, in terms of the mutual definition of surface types, two types of contacts. The first type is surface contact between surfaces, where the initial mutual position in direct contact is set (so-called Adjust to Touch) even if the mutual surfaces have a geometric gap between them. This type of contact was used to set contacts between surfaces. The second type is the contact between the bolt shank and the edge of the sheet metal in the bolt hole (see Figure 7).

For this type of contact, the mutual position of the contact and the target surface is set without adjusting the mutual position, and the gap is defined according to the geometry of the model. Other parameters of the contacts were their stiffnesses. The ANSYS program calculates the contact stiffness of individual contact pairs at the beginning of the solution. The calculated value is usually relatively high for steel models. It often harms the convergence of the calculation, in which the so-called chattering, i.e., a kind of bouncing of mutual surfaces, may occur. There are several ways to avoid this effect. In the described numerical models, the method of contact stiffness reduction was applied. This procedure is common for finite element modelling. The reduction of contact stiffness results in the possibility of penetrating the contact parts more. This does achieve better computational stability of the problem, but at the cost of reducing the accuracy of the results, especially the contact stress. However, with an appropriate choice of reduction, the effect on the results is negligible in terms of the required accuracy and accuracy of the model behaviour. The tested assembly also contained welded components. It was a distribution crossbar and cleats with a welded rib. All welds were simply modelled by so-called bonded contacts, which connected the individual parts with a rigid bond. This idealization of welds has a negligible impact on the results. The stiffness reduction factor for all contacts was chosen as 0.5, based on experience.

### 3.4. Bolt Connections Modelling

**Figure 8:**
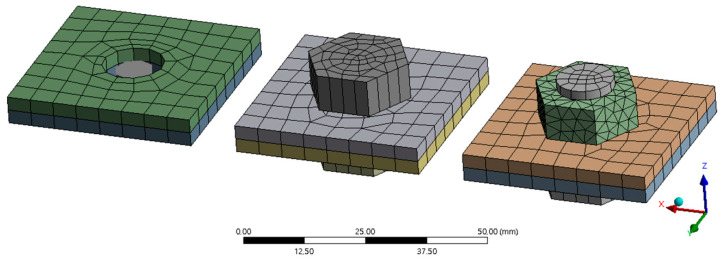
Numerical models of bolted joints—from the left a beam model, a volume with a shell and a full-volume bolt model.

The numerical model of bolt connections was made up of independent parts that were connected by connecting elements. As an effort was made to create the most concise model of the tested assembly, it was necessary to create models of joints that capture as much as possible of the actual behaviour of the joints in the structure. Therefore, numerical simulations of different ways of modelling bolted connections with different degrees of idealization were carried out. The bolts were modelled in four ways: by using the constraint equations defined in ANSYS joints (called joint), by using member elements (BEAM188) and also by combining volume elements (bolt shank) with shells (bolt head and bolt mother). A full-volume model of the bolt was also prepared. Figure 8 shows numerical models of the latter three types of bolt joints connecting two shells. A comparison of the properties was made for the last three types of bolted connections.

The analysis of the results obtained using these models shows that the simplest connection using a member converges computationally best, which is not surprising for a linear model. When comparing the perfectly stiff joint, the connection using the member is slightly softer, but still, a relatively stiff connection compared to the behaviour of the pin. This type of joint was used in the following models at the point of bolting the uprights to the face of the overlap.

### 3.5. Boundary Conditions

**Figure 9:**
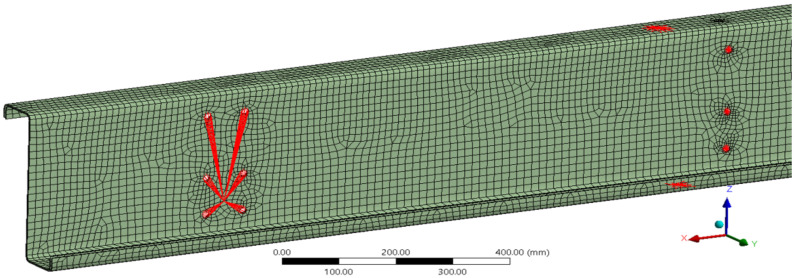
Detail of remote displacement in supporting the ends of beams and placing transverse struts.

To reduce the computational complexity of the task, the end sills were replaced by two spacers (so-called remote displacement) connecting the nodes of the network in the holes at the ends of the beams with a point defined in the axes of the notched joints of the end sills. Their connection to the ends of the purlins is modelled using the MPC algorithm. By this numerical treatment, the shell nodes are tied to a control node located at the centre of rotation of the hinge fitting. The advantage of such a solution is the reduction of the computational complexity of the model, which does not have a significant effect on the resulting values of stress and strain on the part of the model under study (the supra-support region). The bearing is simulated as a non-sliding hinge at the ends of the structure. Schematically, the constraints are shown by red lines in Figure 9.

### 3.6. Material Models

Simulations were carried out to investigate the effect of the different material models used for the thin-walled beams. In the models, this physical nonlinearity is introduced in the form of elastic-plastic material models. The research presented is part of a wider experimental programme involving different plate thicknesses, different truss heights and different spans. Therefore, it was possible to experimentally obtain 57 test steel plates taken from undamaged parts of the purlins after the experiment for tensile tests. A 5% quantile was determined from the results. A yield strength of 440 MPa was obtained for all models based on material analysis [15].

**Figure 10:**
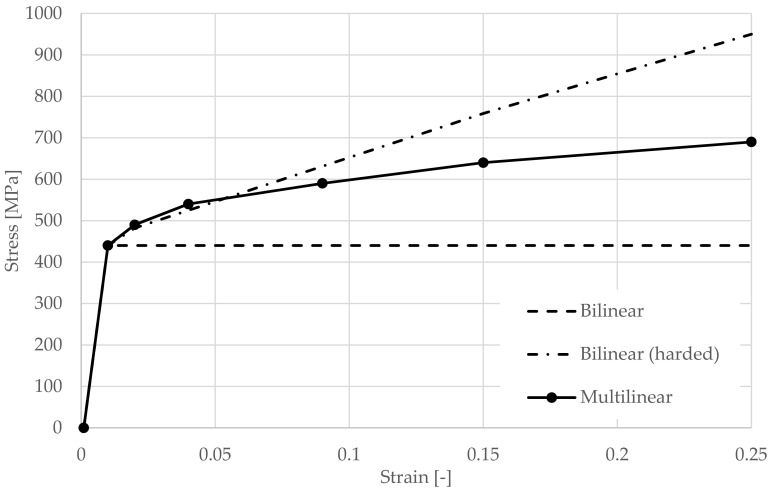
Stress-strain diagram alternatives.

This value is higher than the declared yield strength of the steel used according to the delivery documentation. The bilinear model, the bilinear model with hardening and the multilinear model were studied (see Figure 10). These prepared material models were further used in numerical models purely to test the logical assumption that a multilinear diagram would be the most accurate.

## 4. Results and Discussion

**Figure 11:**
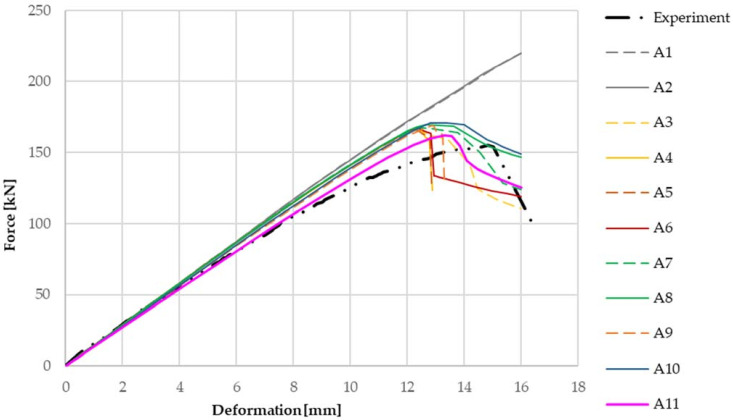
Results from numerical models and experiments using various parameters, with cleat.

**Figure 12:**
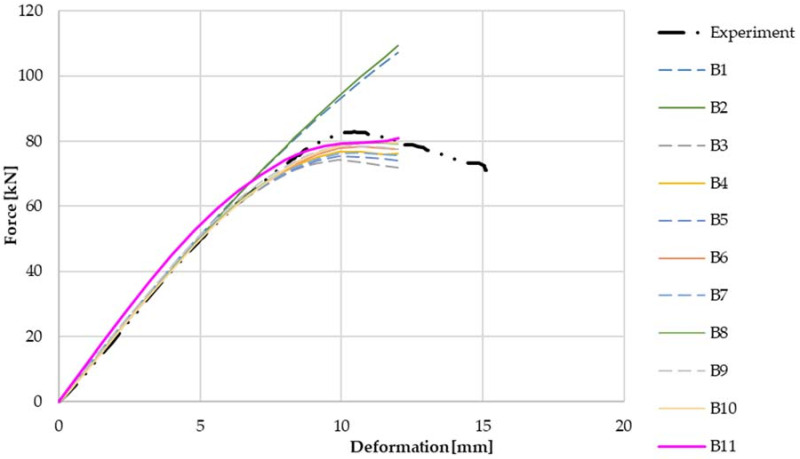
Results from numerical models and experiments using various parameters—without cleat.

As mentioned above, the basic parameters of the models were based on the test Z300 profile with a material thickness of 1.89 mm, a cross-sectional width of 200 mm and a span of 3.0 m. There two sets of models were prepared, one with a reinforcing cleat (see Figure 11) and one without a cleat (see Figure 12). Table 1 shows the parameters of each numerical model without cleats.

All models include geometric nonlinear behaviour, and the contacts were modelled with the application of friction as described above. The table shows different combinations of parameters, and the marking is as follows: “SOLID” when applying element SOLSH190, “SHELL” when applying element SHELL181, “linear” when applying linear material model, “BL” when applying bilinear material model, “BLzp” when applying a bilinear material model with hardening, “ML” when applying a multilinear material model, “joint” when applying joints using the function called joint, and “beams” when applying joints using a small beam element.

From a comparison of the numerical models with the experimental data for the cleat joint assembly shown in Figure 11, the linear model without slip in the joints is much stiffer than the measured values. When material non-linearities are applied, the influence of the formation of plastic areas on thin-walled beams and thus the achievement of the maximum load and the consequent decrease of the force in the critical area is evident. However, this phenomenon alone does not sufficiently capture the actual behaviour of the joint, whose load capacity and rigidity are still lower than those of this model. It follows that the types of joints have a significant effect on the resulting rigidity of the entire detail of the joint. The model with the cleat shows a higher stiffness compared to the experiments and also a higher load-carrying capacity of about 7%. Therefore, the maximum load is also achieved at a lower displacement. This effect is probably due to the large imperfections caused by the displacement of the thin-walled beams during erection. These imperfections have not been taken into account in the numerical models.

From Figure 11 the most matching with the experiment is the numerical model which contains bolt joints that allow slippage between the elements and considers the possibility of pressing the bolt shanks against the walls of the holes given their different dimensions. Furthermore, the graph shows that when comparing the shell and volume models, the results for both types are practically identical, except for the fact that the volume models are slightly less stiff and when using nonlinear material models, they are less tolerable compared to the shell models. Table 2 shows the parameters of each numerical model with cleats.

From a comparison of the working diagrams of the numerical models and the experiments for the joint without the cleat in Figure 12, it is possible to see similar phenomena as described for the previous models with the cleat. The model without cleats shows a higher initial stiffness compared to the experiment, but the resulting capacity is slightly lower, by about 5%. This is the effect of simplified types of joints without slippage on the overall rigidity of the joint. The difference between shell and volume models is a bit more pronounced here. Again, the shell models are found to be stiffer with a higher load capacity.

**Figure 13:**
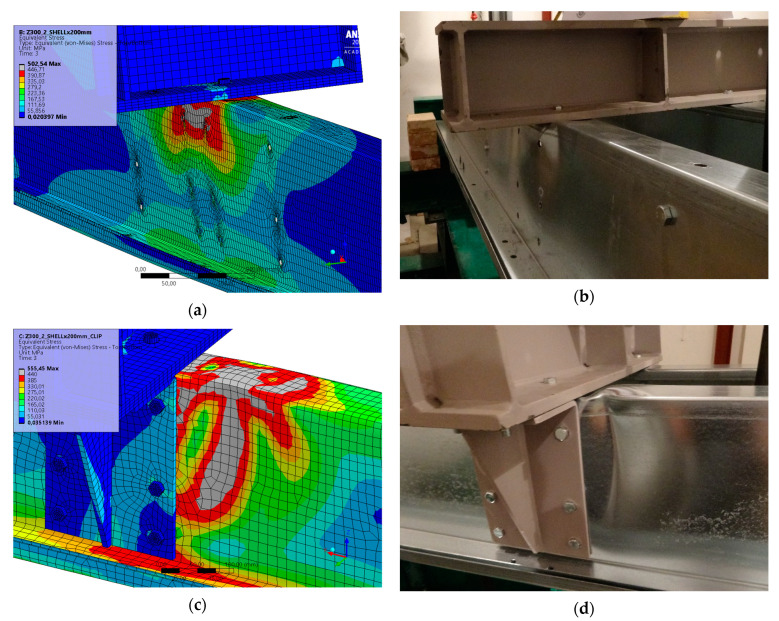
Detail of the purlins and failure mode after the experiment: von Misses stress without cleats (**a**), experiment without cleats (**b**), von Misses stress with cleats (**c**) and experiment with cleats (**d**).

Last, the failure mode of purlins was analysed, which is shown from the model and experiment in Figure 13. In both cases, it was a loss of web stability.

## 5. Conclusions

The possibility of using the modelling of the studied details of roof purlins using the finite element method was confirmed. The results obtained by numerical simulations were in good agreement with the experimental measurements for experimentally verified materials.

Sophisticated numerical models could be used for possible interpolation of intermediate values based on finding their suitability and corresponding to experimental measurements. In this way, it is possible to create numerical models of other connection variants and use their results for structural design. For use in design, experiments with the material with standardized properties would be necessary, especially with a yield strength close to the design value.

In general, it can be said that in the application of more accurate material models and especially types of joints that can simulate effects of shear-out and pull-through of the bolts, the numerical simulation reduces the resulting (higher) theoretical stiffness/bearing capacity of the joint, and thus approaches the actual behaviour of tested joints.

The numerical models exhibit higher stiffness compared to the experiments, mostly at higher loads. This is attributed to the possible influence of imperfections and other design tolerances, for example, in the misalignment of the bolt holes and the resulting differential settlement of the individual parts of the tested assembly during loading.

Further research will be directed at extending the experimental program to include variations in steel sheet thicknesses, purlin heights, spans between supports, and width dimensions of loading parts. Numerical models will then be prepared and verified for all variations. All types of tests should be further compared with the analytical approach from the standard.

## Figures and Tables

**Table 1:** Parameters of numerical models with cleats.

Model No.	Cleats	Element	Material Model	Connection
A1	Yes	SOLSH190	Linear	Joint
A2	Yes	SHELL181	Linear	Joint
A3	Yes	SOLSH190	Bilinear	Joint
A4	Yes	SHELL181	Bilinear	Joint
A5	Yes	SOLSH190	Bilinear (harded)	Joint
A6	Yes	SHELL181	Bilinear (harded)	Joint
A7	Yes	SOLSH190	Multilinear	Joint
A8	Yes	SHELL181	Multilinear	Joint
A9	Yes	SOLSH190	Multilinear	Beam
A10	Yes	SHELL181	Multilinear	Beam
A11	Yes	SHELL181	Multilinear	Shell

**Table 2:** Parameters of numerical models without cleats.

Model No.	Cleats	Element	Material Model	Connection
B1	No	SOLSH190	Linear	Joint
B2	No	SHELL181	Linear	Joint
B3	No	SOLSH190	Bilinear	Joint
B4	No	SHELL181	Bilinear	Joint
B5	No	SOLSH190	Bilinear (harded)	Joint
B6	No	SHELL181	Bilinear (harded)	Joint
B7	No	SOLSH190	Multilinear	Joint
B8	No	SHELL181	Multilinear	Joint
B9	No	SOLSH190	Multilinear	Beam
B10	No	SHELL181	Multilinear	Beam
B11	No	SHELL181	Multilinear	Shell

## Data Availability

No data available.

## References

[1] Nguyen N.L., Jang G.W., Choi S., Kim J., Kim Y.Y. (2018). Analysis of Thin-Walled Beam-Shell Structures for Concept Modeling Based on Higher-Order Beam Theory. Comput. Struct..

[2] Sivapathasundaram M., Mahendran M. (2016). Experimental Studies of Thin-Walled Steel Roof Battens Subject to Pull-through Failures. Eng. Struct..

[3] Kim H., Jang G.W. (2017). Higher-Order Thin-Walled Beam Analysis for Axially Varying Generally Shaped Cross Sections with Straight Cross-Section Edges. Comput. Struct..

[4] Favero Neto A.H., Vieira L.C.M., Malite M. (2016). Strength and Stiffness of Cold-Formed Steel Purlins with Sleeved and Overlapped Bolted Connections. Thin-Walled Struct..

[5] Bernuzzi C., Maxenti F. (2015). European Alternatives to Design Perforated Thin-Walled Cold-Formed Beam-Columns for Steel Storage Systems. J. Constr. Steel Res..

[6] Carbas S., Saka M.P. (2016). Optimum Design of Cold-Formed Thin-Walled Sections Subjected to Axial and Bi-Axial Bending Using Artificial Bee Colony Algorithm. Res. Eng. Struct. Mater..

[7] Zhao C., Yang J., Wang F., Chan A.H.C. (2014). Rotational Stiffness of Cold-Formed Steel Roof Purlin–Sheeting Connections. Eng. Struct..

[8] Brockenbrough R.L. Compression Member Design in the 1996 AISI Specification. Proceedings of the Annual Technical Session, Structural Stability Research Council.

[9] EN 1993-1-3:2009 (2011). Eurocode 3: Design of Steel Structures—Part 1-9: Fatigue. https://standards.iteh.ai/catalog/standards/cen/886bde8e-f737-4e33-ab90-4c73883f6d22/en-1993-1-9-2005.

[10] Dubina D., Ungureanu V., Landolfo R. (2013). Eurocode 3: Design of Steel Structures. Part 1-3 Design of Cold-Formed Steel Structures. https://lib.hpu.edu.vn/handle/123456789/22866.

[11] Bernuzzi C., Simoncelli M. (2017). EU and US Design Approaches for Steel Storage Pallet Racks with Mono-Symmetric Cross-Section Uprights. Thin-Walled Struct..

[12] Becque J., Rasmussen K.J.R. (2013). Stability of Z-Section Purlins Used as Temporary Struts during Construction. J. Struct. Eng..

[13] Shifferaw Y., Woldeyes K., Bitsuamlak G. Stability and Strength Behavior of Thin-Walled Roof-Panel-Purlin System under Wind Loading. Proceedings of the Annual Stability Conference Structural Stability Research Council 2017.

[14] Silva J.M.M., Malite M. (2020). Longitudinally Stiffened Web Purlins under Shear and Bending Moment. Thin-Walled Struct..

[15] Pařenica P., Rosmanit M., Flodr J. (2017). Numerical Modelling of Thin-Walled Purlins Connection to the Supporting Structure. Procedia Eng..

[16] Bathe K.-J. (2008). Finite Element Method. Wiley Encyclopedia of Computer Science and Engineering.

[17] Cheung Y.K., Tham L.G. (2020). Finite Strip Method.

[18] Schafer B.W. Progress on the Direct Strength Method. Proceedings of the International Specialty Conference on Cold-Formed Steel Structures: Recent Research and Developments in Cold-Formed Steel Design and Construction.

[19] Schafer B. (2006). Direct Strength Method (DSM) Design Guide. Design Guide CF06-1.

[20] Quispe L., Hancock G. Direct Strength Method for the Design of Purlins. Proceedings of the International Specialty Conference on Cold-Formed Steel Structures: Recent Research and Developments in Cold-Formed Steel Design and Construction.

[21] ASTRON-R&D Calculated Local Resistances of the Internal Supports for the Overlaped Z Purlins with or without Clip. https://www.astron.nl/research-and-innovation/.

[22] Gutierrez R., Loureiro A., Lopez M., Moreno A. (2011). Analysis of Cold-Formed Purlins with Slotted Sleeve Connections. Thin-Walled Struct..

[23] Gutierrez R., Loureiro A., Reinosa J.M., Lopez M. (2015). Numerical Study of Purlin Joints with Sleeve Connections. Thin-Walled Struct..

[24] Pham C.H., Davis A.F., Emmett B.R. (2014). Numerical Investigation of Cold-Formed Lapped Z Purlins under Combined Bending and Shear. J. Constr. Steel Res..

[25] Obeydi M., Daei M., Zeynalian M., Abbasi M. (2021). Numerical Modeling on Thin-Walled Cold-Formed Steel Clip Angles Subjected to Pull-out Failures. Thin-Walled Struct..

[26] Lehner P., Krejsa M., Pařenica P., Křivý V., Brožovský J. (2019). Fatigue Damage Analysis of a Riveted Steel Overhead Crane Support Truss. Int. J. Fatigue.

[27] EN 1993-1-8 (2005). Design of Steel Structures—Part 1-8: Design of Joints.

[28] ANSYS ANSYS Meshing User’s Guide. https://customercenter.ansys.com/.

[29] Trzepiecinski T. (2019). A Study of the Coefficient of Friction in Steel Sheets Forming. Metals.

